# Assessing Cognitive Impairments in Obstructive Sleep Apnea Patients Using Montreal Cognitive Assessment (MoCA) Scores

**DOI:** 10.7759/cureus.70085

**Published:** 2024-09-24

**Authors:** Darius A Davidescu, Anca Goman, Florica Voita-Mekeres, Aliz I Bradacs, Solea F Sabina Florina, Andrei N Csep, Gheorghe Szilagyi, Alexandru C Motofelea, Lavinia Davidescu

**Affiliations:** 1 Pulmonology, Doctoral School of Biomedical Sciences, University of Oradea, Oradea, ROU; 2 Morphological Disciplines, University of Oradea, Oradea, ROU; 3 Health Sciences, Doctoral School of Biomedical Sciences, University of Oradea, Oradea, ROU; 4 Medicine and Pharmacy, Department of Medicine, University of Oradea, Oradea, ROU; 5 Infectious Disease, Department of Psycho-Neuroscience and Recovery, Faculty of Medicine and Pharmacy, University of Oradea, Oradea, ROU; 6 Surgical Disciplines, Faculty of Medicine and Pharmacy, University of Oradea, Oradea, ROU; 7 Internal Medicine, Victor Babes University of Medicine and Pharmacy, Timisoara, ROU; 8 Pneumology, University of Oradea, Faculty of Medicine and Pharmacy, Oradea, ROU; 9 Pneumology, Hospital of Pneumology, Oradea, ROU

**Keywords:** ess, hypoxemia, moca, polysomnography, sleep apnea

## Abstract

Obstructive Sleep Apnea (OSA) is a chronic condition associated with cognitive impairment and various comorbidities. This prospective study evaluated cognitive deficits in OSA patients and identified clinical factors affecting cognitive function. Seventy-two participants were assessed using polysomnography (PSG) and the Montreal Cognitive Assessment (MoCA). Findings revealed significantly lower MoCA scores in severe OSA patients compared to those with mild or moderate OSA. Severe OSA patients had a median MoCA score of 23.5 (20.0-25.0), indicating more significant cognitive impairment, while those with normal OSA severity had the highest median score of 28.5 (27.8-29.2). Mild and moderate OSA patients had median scores of 26.5 (21.0-28.0) and 25.0 (23.80-26.0), respectively (p < 0.008). Logistic regression showed that ex-smoking status negatively impacted MoCA scores more in the unadjusted model (p = 0.003) than in the adjusted one (p = 0.018). Forced Vital Capacity (FVC) positively correlated with MoCA scores, stronger in the unadjusted model (p < 0.001 vs. p < 0.03). Higher Oxygen Desaturation Index (ODI) correlated with higher MoCA scores while increasing Apnea-Hypopnea Index (AHI) severity correlated with lower MoCA scores in both models. A significant negative correlation was found between age and MoCA score (r = -0.473, p < 0.001), and between MoCA score and AHI (r = -0.350, p < 0.003). This study highlights the need for sensitive cognitive screening tools like MoCA in evaluating OSA patients, linking cognitive impairment closely with OSA severity and other clinical factors.

## Introduction

Obstructive Sleep Apnea (OSA) is a chronic condition that is associated with elevated morbidity rates. Recent studies have reported a prevalence rate of 24% in men aged 30-60 and 9% in women [1]. OSA is characterized by the collapse of the upper airways during sleep, with a duration of more than 10 seconds, accompanied by respiratory efforts, and resulting in more than 3% or 4% desaturation in oxygen, along with sleep disruption [1,2].

The complexity of OSA arises from comorbidities such as respiratory, cardiovascular, metabolic, and neurological disorders. These include hypertension, arrhythmias, stroke risk, insulin resistance, diabetes mellitus, cognitive dysfunction, mood disorders, gastroesophageal reflux disease (GERD), and disruptions in endocrine balance. Nevertheless, the association between these conditions and cognitive impairment has not been extensively investigated [3,4]. In OSA neurocognitive impairment may manifest as excessive daytime sleepiness (EDS) [5], decreased concentration and attention [6-8], memory deficits [8,9], impaired cognitive performance [6], mood changes such as irritability or depression [10,11].

In elderly women, moderate OSA with an AHI > 15 was associated with an increased risk of future development of dementia or mild cognitive impairment [4,12]. Intermittent hypoxia and sleep fragmentation are considered to be the primary mechanisms for cognitive impairment in OSA. Intermittent hypoxia can lead to neuronal damage, particularly in areas involved in memory and executive function, resulting in cognitive impairment [12]. Hypoxia triggers brain inflammation, which can exacerbate cognitive impairment and lead to cerebral microstructural changes, affecting its capacity to meet optimal functional standards [13,14]. Another possible mechanism is sleep fragmentation characterized by frequent awakenings or arousals that occur when the brain responds to a deficiency of oxygen by briefly waking up to reopen the airways. These frequent awakenings result in disrupted sleep patterns and reduced overall sleep quality [15]. The cognitive effects of sleep fragmentation include compromised memory consolidation, as disrupted sleep can impede the process of forming new memories, thus impacting learning and memory functions [16]. Reduced Slow-Wave Sleep, also referred to as deep sleep, is essential for the process of cognitive restoration. OSA disturbs this period of sleep and results in cognitive impairments [17]. Sleep fragmentation can lead to excessive daytime sleepiness, which can hinder the ability to focus and carry out cognitive activities effectively [18]. A comprehensive assessment of cognitive impairment in OSA typically involves a combination of clinical evaluation, questionnaires, and objective tests including the Epworth Sleepiness Scale (ESS), cognitive function questionnaires, neuropsychological testing, and polysomnography (PSG). The Montreal Cognitive Assessment (MoCA) is a commonly employed cognitive screening tool, but it is not widely used as a primary assessment tool for OSA, particularly for identifying mild cognitive impairment and dementia [19,20]. The ESS scale has been extensively used as a tool to evaluate the level of daytime sleepiness in patients diagnosed with obstructive sleep apnea. Despite its widespread application, a considerable proportion of patients with OSA do not exhibit abnormal levels of sleepiness. This limits the effectiveness of ESS in detecting the whole range of cognitive impairment linked with OSA [21]. In contrast, the MoCA instrument has shown superior sensitivity in detecting early neurocognitive alterations [20]. This enhanced sensitivity of MoCA renders it an invaluable tool for identifying early cognitive changes that might otherwise remain undetected. The ability of MoCA to detect subtle neurocognitive changes underscores its importance in the comprehensive evaluation of OSA patients. By employing MoCA, clinicians and researchers can effectively identify the initial signs of cognitive decline, thereby facilitating prompt interventions and improving patient survival. This shift in methodology highlights the need for nuanced and multidimensional approaches in the assessment of cognitive function in OSA, going beyond the constraints of conventional sleepiness measures such as ESS [22]. The present study aimed to establish the correlation between the severity of OSA, cognitive performance, age, BMI, and PSG parameters, and desaturation index, namely minimum, medium, and maximum oxygen saturation levels during sleep, using a comprehensive statistical analysis.

## Materials and methods

Study population

A total of 72 eligible participants were enrolled in this prospective study which was conducted over a period of 8 months from October 1, 2023, to May 30, 2024, in the Sleep Laboratory of the Pneumology Hospital “Dr. Lavinia Davidescu”, Oradea, Romania.

The criteria for inclusion were being over 18 years old, presenting symptoms indicative of OSA, and having undergone a thorough diagnostic assessment using polysomnography (PSG). Among the exclusion criteria were a history of prior or ongoing treatment for OSA within the last 12 months, the presence of acute or chronic conditions that may affect outcomes, women who were pregnant or breastfeeding to avoid physiological confounding factors, recent alcohol consumption within 48 hours prior to enrolment, and use of any substances listed on the National Anti-Doping Agency's "Prohibited List." Significant acute or chronic pulmonary problems were characterized as conditions such as chronic obstructive pulmonary disease (COPD) or severe asthma that could autonomously affect the quality of sleep and levels of oxygen saturation, potentially affecting cognitive outcomes. To reduce confounding factors that may independently impact cognitive function, comorbid conditions such as psychiatric, neurological, and oncological disorders were eliminated. This ensured that any detectable cognitive impairments could be more reliably attributed to OSA.

Data collection and measurement

A comprehensive evaluation of patients was undertaken in this study to diagnose OSA by collecting a broad range of data during the initial diagnostic appointment. Demographic and anthropometric information such as age, sex, height, weight, body mass index (BMI), and place of origin were recorded, along with health-related details including smoking status, symptoms, and comorbidities associated with OSA.

In order to ensure that the cognitive evaluation accurately represented the participants' condition, the MoCA was conducted within 24 hours of the PSG. In order to reduce external distractions and guarantee consistent testing conditions for all participants, all MoCA evaluations were carried out in a quiet and controlled environment. As a precaution against bias, the clinicians who administered the MoCA were unaware of the participants' OSA severity, Apnea-Hypopnea Index (AHI), and other PSG measures. During the MoCA evaluations, investigators were only granted access to non-identifying participant codes. Following the assessment, MoCA scores were promptly recorded and the data was securely entered into a database secured with a password. Polysomnography was conducted continuously throughout the night using standardized equipment. Systematic monitoring and recording of sleep stages, oxygen saturation, and respiratory parameters were conducted for analysis. To ensure impartiality in the statistical analysis of the correlation between cognitive function and OSA parameters, data analysts were kept unaware of participant identity and OSA severity.

Diagnostic tests and assessments

Pulmonary Function Tests (PFT): Measurements of Forced Vital Capacity (FVC), Forced Expiratory Volume during the first second (FEV1), and the FEV1/FVC ratio were used to assess respiratory function.

Sleep and Cognitive Evaluations: The Epworth Sleepiness Scale (ESS) was used to measure daytime sleepiness, while the Montreal Cognitive Assessment (MoCA) was used to evaluate cognitive functions, reflecting the potential cognitive impairment associated with OSA.

Polysomnography (PSG): Conducted using advanced equipment from DeVilbiss Healthcare GmbH, Germany, this key diagnostic component measured the AHI, sleep stages, nocturnal oxygen saturation levels (average, minimum, and maximum), pulse rates, and sleeping positions. Specified sleep technologists and a senior sleep physician analyzed and validated the data in accordance with the standards set by the American Academy of Sleep Medicine [23]. The classification was determined using the Index AHI into three categories: Mild (AHI>5<15/h of sleep), Moderate (AHI>15<29/h), and Severe (AHI>30/h). Among subjects 4 had an AHI<5/h, which is considered normal by the American Academy of Sleep Medicine definition [23].

Ethical compliance

Informed consent was obtained from all participants and the study protocol was approved by the Ethical Committee of the Sleep Laboratory of Pneumology Hospital "Dr. Lavinia Davidescu" in Oradea, Romania (approval no. 353/20.09.2023, dated September 10, 2023). The study strictly adhered to the Declaration of Helsinki. This ensured that all research practices not only met rigorous ethical standards but also safeguarded participant welfare throughout the study.

MoCA scale and ESS

MoCA is a cognitive screening tool consisting of 30 points, distributed across different cognitive domains (20). These set of domains include visuospatial and executive abilities (5 points), naming (3 points), memory (5 points), attention and concentration (6 points), language (3 points), abstraction (2 points), delayed recall (5 points), and orientation skill (6 points). An ideal score of 30 indicates the optimal cognitive function, while scores between 18-25 mean mild cognitive impairment, 10-17 moderate cognitive impairment, and below 10 points indicate severe cognitive impairment.

ESS is a widely used self-report questionnaire designed to assess daytime sleepiness [24]. It consists of eight questions, each asking the individual to rate the likelihood of falling asleep in specific situations or activities, such as watching TV, sitting quietly, or driving a car, on a scale from 0 (no chance of dozing) to 3 (high chance of dozing). The scores from these questions are counted, with the total ESS score ranging from 0 to 24. A score of 10 or higher indicates sleepiness in the patient.

The ESS is commonly used in sleep medicine and research to assess an individual's overall degree of daytime sleepiness. Elevated ESS scores suggest an increased tendency to fall asleep during routine daily activities, which can be indicative of sleep-related disorders such as sleep apnea or narcolepsy [25-27]. It is a valuable tool for healthcare professionals and researchers to evaluate the impact of sleepiness on an individual's daily functioning and to assist in the diagnosing and treatment of sleep disorders [28]. Although the ESS mainly assesses the tendency to sleep, its relevance to cognitive function is underscored by studies that demonstrate the impact of both the quality and quantity of sleep on cognitive processes, as well as the existing correlations between excessive daytime sleepiness and cognitive decline [29]. Considering the potential relationship between sleep, daytime sleepiness, and cognition, ESS can provide a pertinent framework for investigating cognitive function in individuals with OSA. While not directly assessing cognitive function, when used in conjunction with direct cognitive evaluations, it can provide additional insights into the relationships between sleep, sleep disorders (such as OSA), and cognitive outcomes.

Statistical analysis

Data centralization and analysis were conducted using SPSS Statistics for Windows version 26 (IBM Corp., Armonk, USA) and Python version 3.12. The distribution was tested using the Shapiro-Wilk test. Normally, distributed values were reported as mean (SD) and the non-Gaussian values were reported as median (IQR). We employed various statistical tests to confirm correlation relationships, including ANOVA, Tukey's HSD (Honestly Significant Difference), scatter-plotting, and correlation tests using Pearson Correlation. A significance level of p < 0.05 was considered the threshold for statistical significance (alpha of 5%).

To investigate whether the mean MoCA scores significantly differed across various OSA severity levels, a Kruskall-Wallis test was conducted. This test is instrumental in understanding whether there are any statistically significant differences between the means of three or more independent groups.

In the context of our study, the independent variable was the OSA severity level (with four categories: Normal, Mild, Moderate, and Severe), while the dependent variable was the MoCA score.

To explore the linear relationship between AHI and MoCA scores, a Spearman correlation test was employed.

A comprehensive multiple regression analysis was utilized to explore the relationships between cognitive function (MoCA score) and several independent variables, including OSA severity (AHI), age, and BMI. A forward selection approach was adopted, where variables were sequentially added to the model based on their significance in predicting the dependent variable.

VIF (Variance Inflation Factor) and tolerance levels were meticulously checked to ensure that the final model was not afflicted by multicollinearity, safeguarding against inflated standard errors and unstable estimates. Furthermore, the model’s R-squared value was scrutinized to assess the proportion of variance in the dependent variable explained by the model. The significance of individual predictors was gauged through p-values and beta coefficients, providing insights into the relative contribution and directionality of each variable in predicting cognitive function.

## Results

Out of the 72 patients included in our study, 14 (19%) were female and had a median age of 47 years. Most of the participants lived in urban areas (67%) and 39% were smokers (Table 1).

**Table 1:** Demographic measurement of patients Note: Age and BMI (Body mass index) results are presented as n (%) and median (percentile 25th and percentile 75th)

Variable	N (%)
Age	47 (39, 55)
BMI	30 (28, 38)
Gender	
- Female	14 (19%)
- Male	58 (81%)
Residence	
- Urban area	48 (67%)
- Rural area	24 (33%)
Smoking Status	
- Non-smoker	30 (42%)
- Smoker	28 (39%)
- Ex-smoker	14 (19%)

More than half of the patients (56%) reported experiencing both physical and mental exhaustion, while 44% reported the presence of snoring. Sleepiness and dyspnea were less common, impacting 11% of the participants. Insomnia was present in various forms, with 25% having initial insomnia and 29% struggling with maintenance insomnia. Comorbid conditions were notable, with 28% of patients diagnosed with Grade II hypertension and 17% with type 2 diabetes. Pulmonary function tests revealed a median FVC of 81%, while the median AHI was 21 events per hour, reflecting moderate to severe sleep apnea in the subject group. Blood oxygen levels were closely monitored, with a median minimum oxygen saturation (SaO2) of 77% and average SaO2 of 94%. The MOCA scores, which had a median value of 25, indicated varying degrees of cognitive function across the study population (Table 2).

**Table 2:** Baseline characteristics of patients BMI: Body Mass Index, COPD: Chronic Obstructive Pulmonary Disease, AHI: Apnea-Hypopnea Index, FVC: Forced Vital Capacity, SaO2: Oxygen Saturation, MoCA: Montreal Cognitive Assessment. Results are presented as n (%) and median (percentile 25th and percentile 75th).

Characteristic	N (%) or Median (Q1, Q3)	Characteristic	N (%) or Median (Q1, Q3)
Symptomatology n (%)		Comorbidities n (%)	
Physical Fatigue	40 (56%)	Grade I Hypertension	6 (8.3%)
Mental Fatigue	40 (56%)	Grade II Hypertension	20 (28%)
Sleepiness	8 (11%)	Diabetes Mellitus Type II	12 (17%)
Snoring	32 (44%)	COPD	8 (11%)
Dyspnea	8 (11%)	Bronchitis	7 (9.7%)
Initial Insomnia	18 (25%)	Other Comorbidities	15 (21%)
Maintenance Insomnia	21 (29%)	Scores & Measures	
Other Symptoms	14 (19%)	Epworth Score	7.00 (4.75, 9.00)
Pulmonary Function & Vitals		FVC	81 (75, 92)
AHI	21 (13, 49)	Desaturation Index	27 (14, 49)
Min. SaO2	77 (66, 83)	Avg. SaO2	94.0 (91.8, 95.0)
Max. SaO2	100 (99, 100)	Min. Pulse	46 (39, 54)
Max. Pulse	108 (99, 141)	Avg. Pulse	70 (62, 73)
MoCA Score	25.00 (21.00, 27.00)		

In this study, variations in demographic, clinical, and physiological features were noted between female and male participants. The median age of the female participants was 49 years, which was significantly higher than the median age of 46 years for the male participants (p = 0.018). Women also had a higher median BMI (41.5) compared to men (30.0), although this difference was not statistically significant (p = 0.082). Sleepiness, as assessed by the Epworth Score, was similar between genders, with no significant difference (p = 0.071). Cognitive function, assessed by the MOCA Score, showed no gender-related differences (p = 0.668). Lung function, represented by FVC, was slightly lower in females but not significantly so (p = 0.104). However, males had a significantly higher AHI and ODI, indicating more severe sleep apnea and oxygen desaturation (p = 0.021 and p = 0.027, respectively). There was no significant difference in blood oxygenation, including minimum and average SaO2, between genders. However, males were more prone to achieving higher maximum SaO2 levels (p = 0.015). Comparable pulse measures, including minimum, maximum, and average pulse rates, were found in both groups, with no significant differences observed (p > 0.6 for all) (Table 3).

**Table 3:** Comparative Analysis of Demographic, Clinical, and Physiological Variables by Gender in a Patient Cohort ^1 ^Linear Model Anova, ^2^ Chi-Square test, BMI: Body Mass Index, ESS: Epworth Score, FVC: Forced Vital Capacity, AHI: Apnea-Hypopnea Index, ODI: Oxygen Desaturation Index, SaO2: Oxygen Saturation, MoCA: Montreal Cognitive Assessment. Results are presented as n(%) and median (percentile 25th and percentile 75th)

Variable	Female Median (IQR)	Male Median (IQR)	Total Median (IQR)	p-value
Demographics & Anthropometrics				
Age	49.0 (44.5 to 65.0)	46.0 (39.0 to 54.0)	47.0 (39.0 to 55.0)	0.018^1^
BMI	41.5 (29.0 to 46.3)	30.0 (28.0 to 35.2)	30.5 (28.0 to 38.4)	0.082^1^
Clinical Scores				
Epworth Score	7.0 (7.0 to 11.5)	7.0 (4.0 to 9.0)	7.0 (4.8 to 9.0)	0.071^1^
MOCA Score	25.0 (22.0 to 27.0)	24.0 (21.2 to 27.0)	25.0 (21.0 to 27.0)	0.668^1^
Lung Function				
FVC	78.0 (73.2 to 87.5)	82.0 (78.0 to 92.0)	81.0 (74.8 to 92.0)	0.104^1^
AHI	14.5 (12.3 to 18.4)	23.7 (13.6 to 51.5)	21.1 (12.5 to 49.3)	0.021^1^
ODI	16.2 (14.0 to 25.2)	29.6 (14.3 to 54.9)	26.7 (14.3 to 49.2)	0.027^1^
Blood Oxygenation				
Min. SaO2	79.0 (67.5 to 82.0)	77.0 (67.0 to 84.0)	77.0 (66.5 to 83.2)	0.531^1^
Avg. SaO2	93.0 (91.5 to 94.0)	94.0 (92.0 to 95.0)	94.0 (91.8 to 95.0)	0.624^1^
Max. SaO2				0.015^2^
97	0 (0.0%)	2 (3.4%)	2 (2.8%)	
98	4 (28.6%)	2 (3.4%)	6 (8.3%)	
99	2 (14.3%)	20 (34.5%)	22 (30.6%)	
100	8 (57.1%)	34 (58.6%)	42 (58.3%)	
Pulse Measures				
Min. Pulse	46.0 (38.2 to 55.5)	46.0 (39.0 to 54.0)	46.0 (39.0 to 54.0)	0.628^1^
Max. Pulse	116.0 (105.8 to 123.0)	106.0 (98.0 to 153.0)	107.5 (98.8 to 141.0)	0.628^1^
Avg. Pulse	68.0 (64.2 to 72.2)	71.0 (62.0 to 73.0)	70.0 (62.0 to 73.0)	0.909^1^

The study comprised 72 patients who were classified into four separate groups according to their Apnea-Hypopnea Index (AHI): Normal (N = 4, 5.6%), Mild (N = 20, 28%), Moderate (N = 20, 28%), and Severe (N = 28, 39%). Significant variations in several key characteristics were observed when comparing different parameters across these AHI categories.

Patients in the Moderate and Severe AHI categories were generally older, with a median age of 57 years and 47 years, respectively, compared to 42 years in both the Normal and Mild groups (p = 0.031). A significant gender imbalance was observed, with all patients in the Normal and Severe groups being male, while females were exclusively found in the Mild and Moderate categories (p < 0.001) (Table 4).

**Table 4:** Comparation of parameters of AHI categories BMI: Body Mass Index; ESS: Epworth Sleepiness Scale; FVC represents Forced Vital Capacity, a measure of lung function; ODI is the Oxygen Desaturation Index, which measures the number of times per hour of sleep that the blood's oxygen level drops by a certain degree from baseline; SaO2 refers to the saturation of peripheral oxygen, with the Minimum indicating the lowest recorded oxygen saturation, Mean showing the average oxygen saturation, and Maximum representing the highest recorded oxygen saturation; Pulse Min/Max/Mean denotes the minimum, maximum, and mean pulse rates recorded. MoCA stands for the Montreal Cognitive Assessment, a cognitive screening test. The results are presented as n(%) and median (percentile 25th and percentile 75th).

Characteristic	Normal (N = 4, 5.6%)	Mild (N = 20, 28%)	Moderate (N = 20, 28%)	Severe (N = 28, 39%)	p-value
Age	42 (37, 47)	42 (39, 49)	57 (46, 64)	47 (39, 55)	0.031
Gender					<0.001
Female	0 (0%)	8 (40%)	6 (30%)	0 (0%)	
Male	4 (100%)	12 (60%)	14 (70%)	28 (100%)	
BMI (kg/m2)	42 (32, 51)	29 (27, 36)	29 (28, 40)	34 (28, 38)	0.2
Smoking status					0.29
Non-smoker	4 (100%)	10 (50%)	8 (40%)	8 (29%)	
Smoker	0 (0%)	6 (30%)	8 (40%)	14 (50%)	
Ex-smoker	0 (0%)	4 (20%)	4 (20%)	6 (21%)	
Provenience					0.43
Urban	4 (100%)	14 (70%)	14 (70%)	16 (57%)	
Rural	0 (0%)	6 (30%)	6 (30%)	12 (43%)	
ESS	5.00 (4.00, 6.00)	6.00 (4.00, 7.00)	9.00 (3.00, 10.00)	7.00 (5.00, 9.00)	0.14
FVC	80 (78, 82)	92 (86, 103)	84 (78, 92)	76 (67, 81)	<0.001
ODI	4 (2, 6)	12 (8, 14)	21 (18, 28)	57 (44, 69)	<0.001
SaO2 Minimum	86 (84, 88)	85 (79, 88)	78 (70, 79)	68 (55, 72)	<0.001
SaO2 Mean	95.0 (94.0, 96.0)	94.5 (94.0, 95.0)	93.5 (93.0, 95.0)	92.0 (87.0, 94.0)	<0.001
SaO2 Maximum					0.006
97%	0 (0%)	0 (0%)	2 (10%)	0 (0%)	
98%	2 (50%)	0 (0%)	4 (20%)	0 (0%)	
99%	0 (0%)	8 (40%)	6 (30%)	8 (29%)	
100%	2 (50%)	12 (60%)	8 (40%)	20 (71%)	
Pulse Min	46 (39, 53)	49 (44, 56)	46 (36, 54)	43 (36, 55)	0.21
Pulse Max	144 (106, 181)	120 (100, 163)	106 (98, 120)	104 (96, 153)	0.28
Pulse Mean	72 (70, 74)	64 (62, 71)	70 (59, 71)	72 (62, 74)	0.16
MoCA Score	28.5 (27.8, 29.2)	26.5 (21.0, 28.0)	25.0 (23.8, 26.0)	23.5 (20.0, 25.0)	0.008

The groups exhibited some variation in BMI showed some variation across the groups, with the highest median BMI observed in the Normal category at 42 kg/m². However, this difference was not statistically significant (p = 0.2). Smoking status also differed among the groups, with a higher prevalence of smokers in the Severe category (50%) compared to none in the Normal group, although this difference did not reach statistical significance (p = 0.29). No significant difference in provenience was observed, whether urban or rural, across the AHI categories (p = 0.43). ESS scores were highest in the Moderate group (median 9.00), reflecting greater daytime sleepiness, although the differences across groups were not statistically significant (p = 0.14). Pulmonary function, as assessed by FVC, exhibited a distinct correlation with severity of AHI. The normal group had the highest FVC (median 80%), while the Severe category had the lowest median at 76%. This difference was statistically significant (p < 0.001). The ODI increased progressively with AHI severity. Patients in the Severe category had the highest desaturation events (median 57) whereas the Normal group had only 4 (p < 0.001). Blood oxygenation levels also varied significantly across groups. The lowest SaO2 was found in the Normal group (median 86%) and lowest in the Severe group (median 68%), while the mean SaO2 followed a similar trend (p < 0.001 for both). Maximum SaO2 showed significant differences as well, with a higher proportion of patients in the Normal and Mild groups achieving near-normal oxygen saturation levels (p = 0.006). No significant differences were observed in pulse measures, including minimum, maximum, and mean pulse rates, among groups (p > 0.2 for all). With increasing severity of AHI, cognitive function, as assessed by the MOCA Score, decreased. The Severe group had the lowest median MOCA Score of 23.5 compared to 28.5 in the Normal group (p = 0.008). Overall, the data suggest that as AHI severity increases, there is a proportional rise in age, oxygen desaturation events, and a decline in cognitive function. This underscores the substantial influence of severe sleep apnea on various health parameters (Table 4).

Cognitive function across OSA severity levels

The study comprised 72 patients who were classified into four individual groups according to their Apnea-Hypopnea Index (AHI): Normal (N = 4, 5.6%), Mild (N = 20, 28%), Moderate (N = 20, 28%), and Severe (N = 28, 39%). The comparison of various parameters across these AHI categories revealed significant differences in several key characteristics.

The median age of patients in the Moderate and Severe AHI categories was of 57 years and 47 years, respectively, compared to 42 years in both the Normal and Mild groups (p = 0.031). A significant gender imbalance was observed, with all patients in the Normal and Severe groups being male, while females were exclusively found only in the Mild and Moderate categories (p < 0.001).

The group exhibited some variations in BMI, with the Normal category having the highest median BMI of 42 kg/m². However, the divergence was not statistically significant (p = 0.2). Differences in smoking status were observed among groups, with a higher prevalence of smokers in the Severe category (50%) compared to no smokers in the Normal group. Nevertheless, this difference did not reach statistical significance (p = 0.29).

Provenience, whether urban or rural, did not significantly differ across the AHI categories (p = 0.43). The Epworth Sleepiness Scale (ESS) scores were highest in the Moderate group (median 9.00), reflecting greater daytime sleepiness, although differences across groups were not statistically significant (p = 0.14).

Pulmonary function, as assessed by Forced Vital Capacity (FVC), showed a clear trend with severity of AHI. Patients in the Normal group had the highest FVC (median 80%), while those in the Severe category had the lowest (median 76%), with the difference being statistically significant (p < 0.001). The Oxygen Desaturation Index (ODI) increased progressively with AHI severity, with patients in the Severe category experiencing the highest desaturation events (median 57) compared to only four in the Normal group (p < 0.001).

Blood oxygenation levels also varied significantly across the groups. Minimum SaO2 was highest in the Normal group (median 86%) and lowest in the Severe group (median 68%), while the mean SaO2 followed a similar trend (p < 0.001 for both). Significant differences were observed in Maximum SaO2, with a greater percentage of patients in the Normal and Mild groups achieving oxygen saturation levels close to normal (p = 0.006).

No significant differences were observed in pulse measures, including minimum, maximum, and mean pulse rates, among groups (p > 0.2 for all). Cognitive function, as assessed by MOCA Score, declined with the increase in AHI severity. The Severe group had the lowest median MOCA Score of 23.5 compared to 28.5 in the Normal group (p = 0.008).

Overall, the data indicate that as AHI severity increases, there is a proportional rise in age, occurrence of oxygen desaturation, and a deterioration in cognitive function. This underscores the influence of severe sleep apnea on various health parameters.

**Figure 1:**
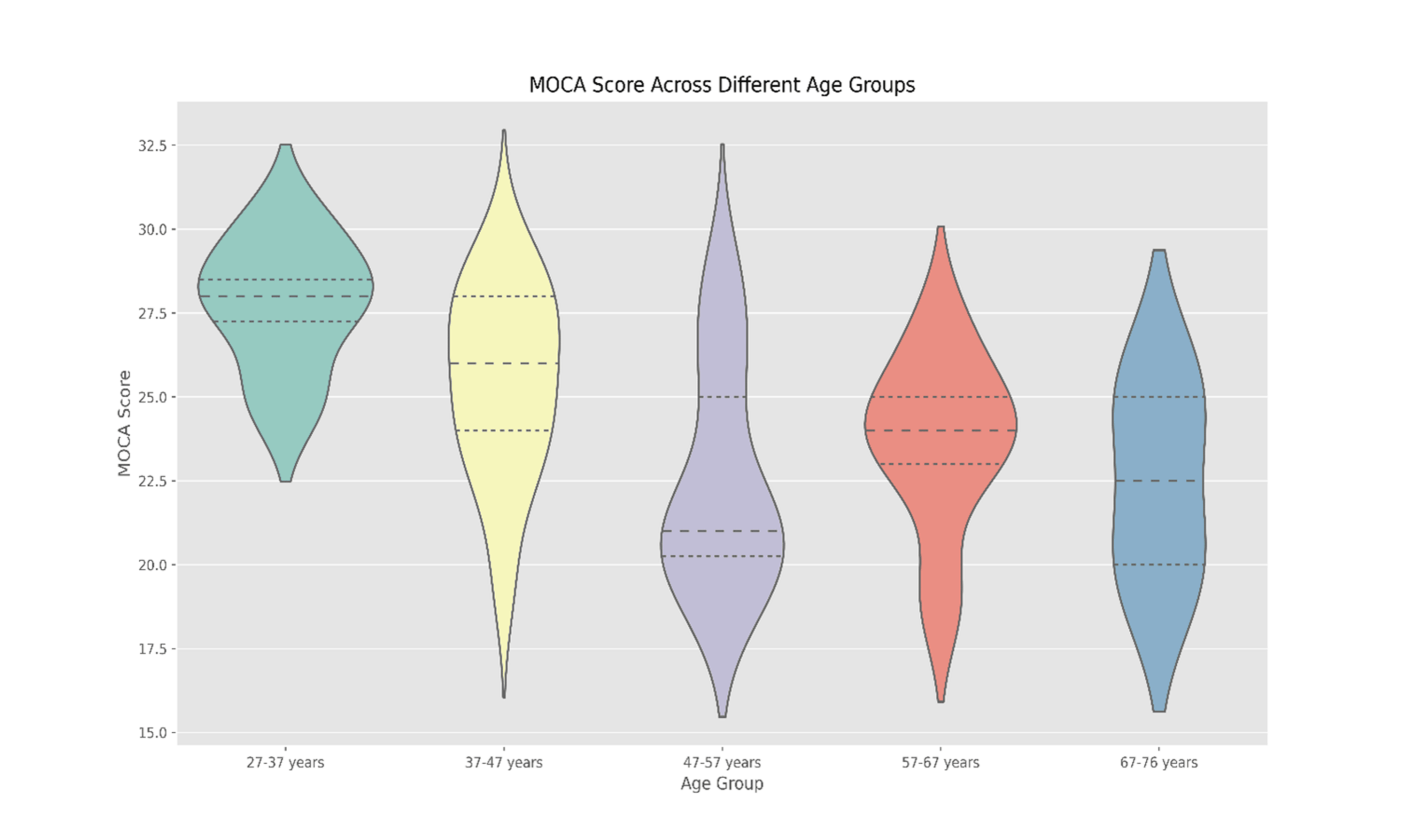
Distribution of MoCA scores across age groups within OSA severity levels MoCA: Montreal Cognitive Assessment score; OSA: Obstructive Sleep Apnea

**Figure 2:**
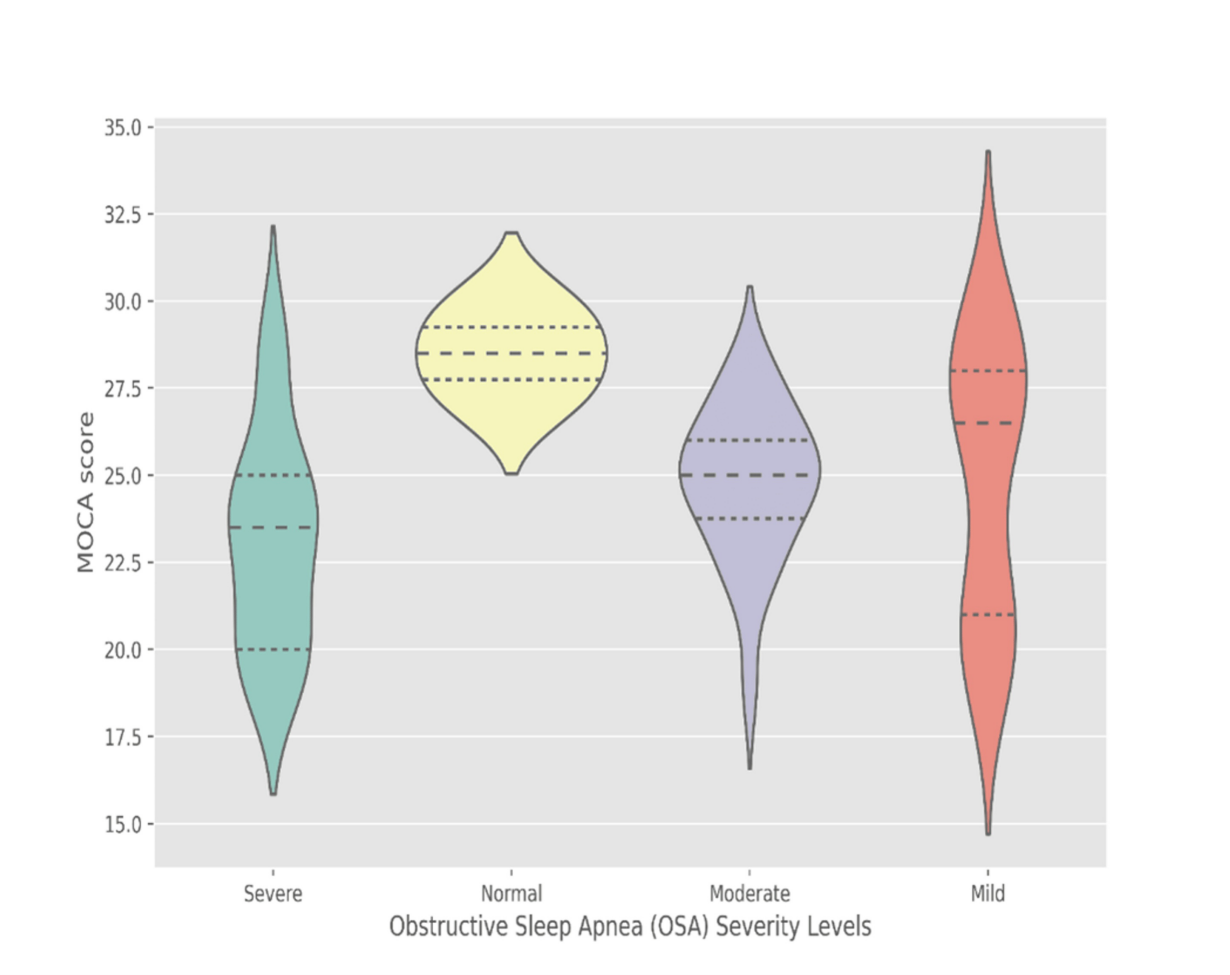
Distribution of MoCA scores across OSA Severity Levels MoCA: Montreal Cognitive Assessment score; OSA: Obstructive Sleep Apnea

The median MoCA scores were as follows: 26.2 for the 27-37 years group, 28.0-29.2 for 37-47 years, 23.9-28.0 for 47-57 years, 23.0-25.0 for 57-67 years, and 20.0-25.0 for 67-76 years. The Kruskal-Wallis test (p < 0.01) revealed statistically significant differences in cognitive function between age groups, with cognitive performance generally declining with increasing age (Figure 1). The MoCA score serves as a gauge for cognitive function, with lower scores indicating heightened cognitive impairment. Our data suggests a discernible pattern: as OSA severity escalates, a decline in MoCA scores is observed, hinting at a potential negative correlation between OSA severity and cognitive function. It is important to note that all four participants who had Normal OSA severity had a MoCA score of 27 (Figure 2).

**Figure 3:**
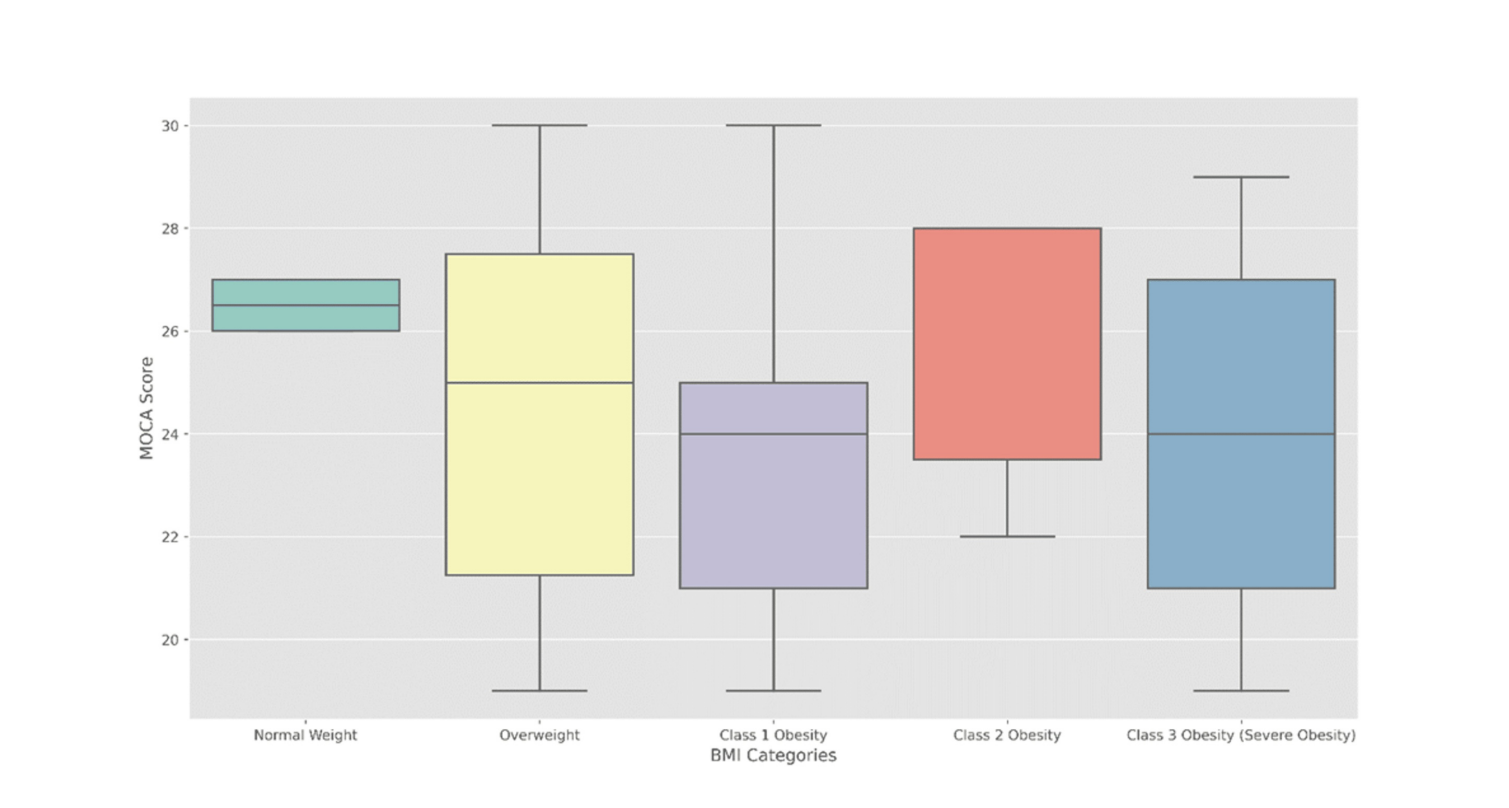
Distribution of MoCA scores across BMI groups within OSA severity levels MoCA: Montreal Cognitive Assessment score; OSA: Obstructive Sleep Apnea

In each group, the median MoCA values were as follows: class 1 obesity had a mean value of 24.0 (IQR 21.0 to 25.0), class 2 obesity had a median value of 28.0 (IQR 23.5 to 28.0) and obesity class 3 (severe obesity) had a median value of 24.0 (IQR 21.0 to 27.0), for normal weight the median was 26.5 (IQR 26.0 to 27.0) and for overweight the median was 25.0 (IQR 21.2 to 27.5). Statistical analysis using the Kruskal-Wallis rank sum test yielded a p-value of 0.33, indicating that there are no significant differences in MoCA values ​​between weight categories as measured by the MoCA in this sample (Figure 3).

Among patients with severe OSA, the median MoCA score was 23.5 (IQR: 20.0, 25.0), suggesting a more pronounced cognitive impairment in this group. In contrast, patients classified as having normal OSA severity exhibited the highest median MoCA score of 28.5 (IQR: 27.8, 29.2), indicating a better cognitive function. Those with mild and moderate OSA exhibited intermediate MoCA scores, with medians of 26.5 (IQR: 21.0, 28.0) and 25.0 (IQR: 23.8, 26.0), respectively p<0.008 (Figure 2).

**Figure 4:**
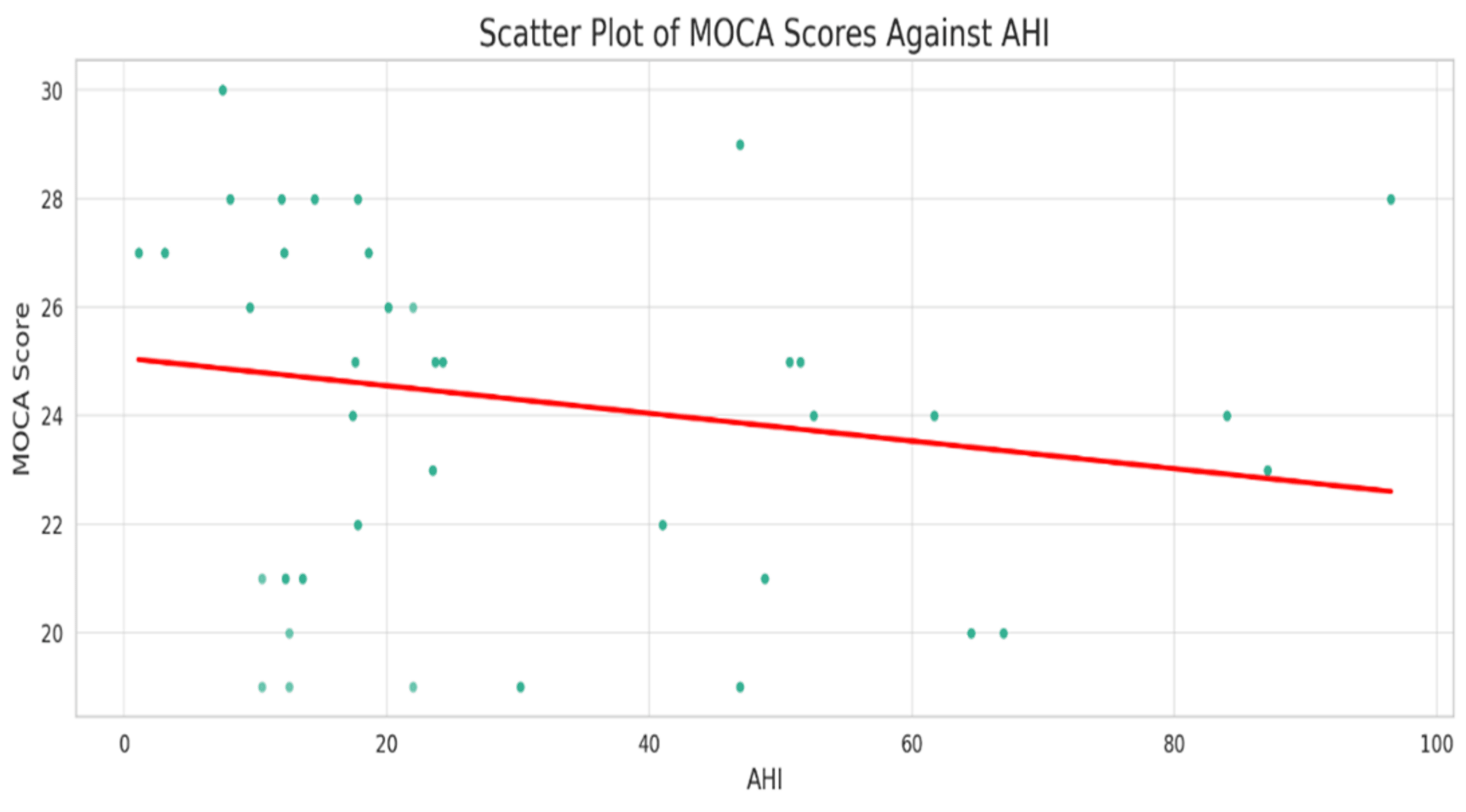
Scatter plot of MoCA scores against AHI. The red line represents a fitted linear regression line that shows the general trend of MoCA scores as AHI increases. MoCA: Montreal Cognitive Assessment score; OSA: Obstructive Sleep Apnea; AHI: Apnea-Hypopnea Index

In Figure 4, a scatterplot is used to illustrate the relationship between AHI and MoCA scores. The scatterplot shows a dispersed, non-linear pattern with a non-significant correlation coefficient, r = -0.202, p = 0.089.

The unadjusted logistic regression model showed a 44% variation, while the adjusted model was able to achieve an R2 of 0.48, showing a modest increase. The regression analysis examines the influence of different predictors on the MoCA score using two models: one using adjusted variables (accounting for age and gender) and one unadjusted. Both models indicate a substantial adverse effect on ex-smokers, with a stronger effect observed in the unadjusted model (0.018 vs 0.003), as shown in Table 5. Both models show a robust positive correlation between FVC and MoCA scores, with a much stronger relationship in the unadjusted model p<0.03 compared to the adjusted model p<0.001. A higher ODI is associated with higher MoCA scores in both models. Increasing severity of AHI (from normal to severe) correlates with significantly lower MoCA scores across both models.

**Table 5:** Comparison of adjusted and unadjusted regression models for the MoCA score The adjusted model accounts for age and gender, while the unadjusted model does not. Key predictors include Smoking Status, ESS (Epworth Sleepiness Scale), FVC (Forced Vital Capacity), ODI (Oxygen Desaturation Index), and AHI (Apnea-Hypopnea Index). ^*^Significant p-values (p < 0.05) to indicate predictors with a statistically significant impact on the MoCA (Montreal Cognitive Assessment) score.

Predictor	Estimate (Adjusted)	SE (Adjusted)	t value (Adjusted)	P (Adjusted)	Estimate (Unadjusted)	SE (Unadjusted)	t (Unadjusted)	p (Unadjusted)
Intercept	26.2107	7.1562	3.663		15.9761	2.9546	5.407	
Smoking status: Smoker – Non-Smoker	-0.6326	0.801	-0.79	0.433	-0.8173	0.7704	-1.061	0.293
Smoking status: Ex-smoker – Non-smoker	-2.305	0.9475	2.433	0.018^*^	-2.819	0.8955	-3.148	0.003^*^
ESS	-0.126	0.1131	1.114	0.27	-0.0263	0.1005	-0.262	0.794
FVC	0.1202	0.0551	2.182	0.033^*^	0.1531	0.0324	4.718	
ODI	0.0746	0.0332	2.25	0.028^*^	0.0934	0.0287	3.252	0.002^*^
AHI Mild – Normal	-6.1119	1.8844	3.243	0.002^*^	-5.3422	1.51	-3.538	
AHI: Moderate – Normal	-4.65	1.9897	2.337	0.023^*^	-5.1215	1.5455	-3.314	0.002^*^
AHI: Severe – Normal	-7.8423	2.4407	3.213	0.002^*^	-8.9723	2.0599	-4.356	

## Discussion

The main objective in this study was to examine the impact of OSA on cognitive function using the MOCA instrument and accounting for confounding factors such as age and gender. In both models, the study revealed association between OSA and MOCA scores, showing a significant association between significantly lower MOCA scores and higher AHI severity (from normal to severe).

Patients with OSA may experience a decline in cognitive function over time, which can lead to substantial changes in their daily life, including difficulties with memory, attention, and decision-making. MOCA scale is a highly effective tool for assessing cognitive function and it is commonly employed in the evaluation of obstructive sleep apnea. One notable finding was the evident differentiation in MoCA scores among various OSA severity categories. The statistical analysis of variance (ANOVA) tests highlight the impact of OSA on cognitive function. This finding is consistent with existing literature that emphasizes the detrimental effects of recurrent nocturnal hypoxemia and sleep fragmentation on cognitive abilities [11,12,15]. These findings underscore the necessity of early diagnosis and intervention, especially in older adults who may be more vulnerable to cognitive decline [1,6].

Within our regression models, age was found to be a substantial factor influencing cognitive function. This correlation between increasing age and cognitive decline is well-documented, but its intricate interaction within the population of patients with OSA presents an intriguing subject for further research. Early diagnosis and intervention are crucial, particularly for older adults at risk of cognitive vulnerabilities [1,6,30]. The negative relationship between age and MoCA score (r=0.426, p<0.01) aligns with established literature, confirming that advancing age is often accompanied by a decline in cognitive faculties (12). Conversely, the substantial influence of oxygen saturation during the night on cognitive function indicates that compromised respiratory function during sleep may impair cognitive abilities, warranting further investigation into the link between respiratory health and cognitive performance [13,14]. Intermittent hypoxia in obstructive sleep apnea (OSA) leads to cognitive decline by causing oxidative stress and brain inflammation, consistent with previous studies. The diminished cognitive scores seen in OSA patients can be attributed to damage to the hippocampus and prefrontal cortex, which are crucial regions for memory and executive function. This underscores the importance of early intervention.

Obesity serves as the most significant risk factor for OSA and plays a crucial role in predicting cognitive impairment. Our findings show a negative correlation between the total MOCA score and BMI (r = -0.025, p > 0.05). Fragmentation of sleep disturbs the essential deep sleep phases required for cognitive recovery. Indicated by progressively lower MOCA scores with increasing OSA severity, our data demonstrates how frequent arousals in OSA patients impair attention, memory, and executive function. Optimizing sleep quality is crucial for maintaining cognitive function during treatment of OSA. Furthermore, in the Kurskall Wallis analysis, there was no significant difference observed between BMI groups (p = 0.33). Several studies have previously investigated the connection between obesity markers, including BMI, fat mass, abdominal fat, and waist-to-hip ratio, and cognitive impairment [31-33] . The combination of obesity and OSA may speed up the progression of Alzheimer's disease due to their strong association and potential involvement in neurodegenerative processes [34]. A study also found that untreated OSA can worsen conditions like hypertension and diabetes, further increasing the risk of cognitive decline. This means that OSA not only occurs alongside these health issues but may also make them worse, leading to a higher chance of developing dementia. This highlights the importance of treating OSA early to reduce these risks and protect brain health [35].

Untreated OSA is linked to comorbidities and a higher risk of severe medical conditions [36]. Among our study population, hypertension was present in 26 patients (36.3%), and Type II diabetes in 20 patients (20.8%). A study of 1,440 participants found that midlife systolic hypertension, persisting into later life, was associated with a 1.6- to 2-fold increased risk of dementia over an 18-year follow-up [37]. Possible pathways connecting hypertension to dementia include small vessel disease, major artery atherosclerosis, and cardiac dysfunction leading to cerebral hypoperfusion [37]. Those with type 2 diabetes have a 1.6-fold higher risk of dementia, according to a meta-analysis involving 283 million individuals [38]. 

The study group comprised 39% current smokers and 19% ex-smokers. A Chinese study involving smokers, ex-smokers, and non-smokers aged 20-60 found that the combination of OSA and chronic smoking results in greater cognitive impairment than smoking alone [39]. 

The study also highlighted the association between living environment and cognitive function. Residing in a rural environment was associated with lower MoCA scores compared to urban settings. This could be due to factors such as reduced access to healthcare, lower levels of social engagement, and environmental aspects. The influence of these elements on cognitive function merits deeper exploration to understand how living environments mediate or modulate cognitive outcomes in OSA patients [11].

Integrating the ESS into our analysis recognized its potential interplay with cognitive function. Although ESS is not a direct measure of cognition, its scores can provide context when exploring the broader implications of sleep quality and quantity on cognitive processes. The association between OSA and cognitive impairment, as underscored by numerous studies, emphasizes the need for early detection and management to mitigate potential cognitive consequences [8,9].

The significant predictive value of age on MoCA scores, consistent with prevailing literature, highlights the exacerbation of age-related cognitive decline by sleep disturbances such as OSA. The non-significant role of BMI in predicting MoCA scores aligns with heterogeneous findings in existing research, suggesting that the relationship between obesity, OSA, and cognitive function may be modulated by various factors, including age, gender, and comorbidities [12].

Given the significant association between OSA severity and cognitive function, our findings underscore the importance of cognitive screening as part of OSA patient evaluations. A multidimensional approach in managing OSA, addressing both sleep disturbances and potential cognitive implications, is essential, particularly in older adults [12].

Lastly, the significant association between rural living and decreased MoCA scores calls for targeted strategies to support cognitive health in rural populations. Enhancing access to healthcare services, promoting social engagement, and improving environmental conditions in rural areas could mitigate these negative effects [11]. This comprehensive approach to managing OSA can ultimately improve patient outcomes [11].

Future research is warranted to explore possible interaction effects or the role of other clinical, behavioral, or environmental factors. Longitudinal studies would provide deeper insights into the trajectory of cognitive changes in relation to OSA and its management. Exploring mechanisms underlying the relationship between OSA, BMI, age, and cognitive function, such as inflammatory markers, oxidative stress, and metabolic parameters, may reveal potential therapeutic targets to improve cognitive impairments in OSA patients [13,14].

This study has a number of limitations that must be recognized. Initially, the rather limited sample size could diminish the statistical power of the findings and confine the applicability of the results to larger populations. The use of a larger sample size would yield more robust conclusions. Furthermore, the study was carried out exclusively at one particular center, so potentially restricting the generalization of the results to other geographic areas or clinical environments. Multi-center studies would facilitate the validation of results across varied populations. Furthermore, the inclusion of self-reported data, namely about smoking status and sleepiness (measured by the Epworth Sleepiness Scale), may introduce bias or inaccuracies, since participants may either underestimate or overestimate their behaviors. The implementation of objective measures or more rigorous controls in future studies would effectively reduce these biases. Finally, although we employed a backward regression method to consider important variables, there may still exist additional unquantified confounding elements, such as lifestyle habits or unreported medical conditions, that could impact cognitive function in patients with Obstructive Sleep Apnea.

Future investigations should prioritise the examination of the enduring effects of treatments like Continuous Positive Airway Pressure (CPAP) therapy on cognitive abilities in individuals diagnosed with Obstructive Sleep Apnea (OSA). The efficacy of CPAP therapy in alleviating the adverse consequences of intermittent hypoxia and sleep fragmentation has been demonstrated. However, further investigation is required to fully comprehend its capacity to reverse or avert cognitive deterioration in the long run. Furthermore, future studies should thoroughly examine the particular cognitive areas that are most impacted by obstructive sleep apnoea (OSA), such as memory, attention, executive function, and visuospatial abilities, in order to pinpoint specific treatment approaches. Analysing the impact of both the severity of obstructive sleep apnoea (OSA) and the length of therapy on different cognitive domains will offer experts useful information for optimising treatment strategies to enhance cognitive function and overall quality of life in OSA patients. Increasing the scope of these studies to encompass larger and more varied populations will further improve the applicability of the results and facilitate the creation of tailored treatments.

## Conclusions

This study highlights the significant impact of OSA severity on cognitive function, with severe OSA patients exhibiting the lowest MoCA scores. Factors such as ex-smoking status, lower Forced Vital Capacity, and higher Apnea-Hypopnea Index are closely associated with greater cognitive impairment. The findings underscore the need for routine cognitive screening, using tools such as MoCA assessment, in the evaluation and management of OSA patients in order to identify and address cognitive deficits early.
